# Influence of Titanium Surface Porosity on Adhesive Strength of Coatings Containing Calcium Silicate

**DOI:** 10.3390/ma13204493

**Published:** 2020-10-10

**Authors:** Ivan Zhevtun, Pavel Gordienko, Yury Kulchin, Alexander Nikitin, Dmitry Pivovarov, Sofia Yarusova, Andrey Golub, Pavel Nikiforov, Vadim Timchenko

**Affiliations:** 1 Institute of Chemistry, Far Eastern Branch, Russian Academy of Sciences, Vladivostok 690022, Russia; pavel.gordienko@mail.ru (P.G.); yarusova_10@mail.ru (S.Y.); golub@ich.dvo.ru (A.G.); 2 Institute of Automation and Control Processes, Far Eastern Branch, Russian Academy of Sciences, Vladivostok 690041, Russia; kulchin@iacp.dvo.ru (Y.K.); anikitin@iacp.dvo.ru (A.N.); diamante_gdi-1@mail.ru (D.P.); vadim@dvo.ru (V.T.); 3 Department of Ecology and Ecological Problems of Chemical Technology, Vladivostok State University of Economics and Service, Vladivostok 690014, Russia

**Keywords:** titanium alloy, silicate coating, wollastonite, lead silicate binder, laser cladding, titanium carbide, surface porosity, adhesive strength

## Abstract

Titanium-based alloys are widely used in aerospace engineering. They have good mechanical and corrosion properties but, in some cases, the material itself or the coating should meet some additional requirements. For example, it may be a requirement of high reflectance to provide effective temperature control. Wollastonite is a promising component for reflective coatings because it improves both their whiteness and mechanical properties. This paper presents the results of studying the composition, the structure and the adhesive strength of wollastonite-containing silicate coatings to titanium substrate. The surface of titanium samples was pre-treated by laser cladding with TiC and etching to provide surface porosity. It has been shown that such treatment allows a significantly increase in the adhesive strength of the coating to the substrate. A decrease in the adhesive strength was observed on titanium samples with an excess of TiO_2_ on the surface. This is caused by the formation of crystalline PbTiO_3_ at the interface.

## 1. Introduction

Titanium and its alloys are widely applied as construction materials in many fields, including aerospace engineering. The use of titanium-based materials is caused by the attractive combination of properties such as high strength, low density and high corrosion resistance. Pure titanium and some its alloys do not embrittle even in liquid helium, while another titanium alloys may be applied at temperatures up to 600 °C. In all temperature ranges, they have higher specific strength than many other construction materials [1,2].

While using titanium alloys for creating spacecrafts, special coatings with high reflection coefficients may be required to provide better temperature control. Common pigments for such coatings are metal oxides such as ZnO, TiO_2_, Al_2_O_3_, ZrO_2_ or spinels such as Zn_2_TiO_4_, MgAl_2_O_4_ [3,4,5]. Silicate materials or organic compounds are used as a binder [6,7]. Some additional information on temperature-controlling coatings can be found in [8].

Natural silicates and their synthetic analogues are widely used to produce construction materials, highly selective sorbents, pigments for various composite materials and coatings [9,10,11]. Wollastonite CaSiO_3_ is a promising and interesting representative of this class of compounds. Due to its high melting point, low thermal coefficient of linear expansion and high strength of needle-shaped crystals which are able to reinforce different materials, wollastonite is a useful component in manufacturing dry building mixes, glasses, ceramics and polymer materials [12]. Synthetic wollastonite containing the minimum amount of impurities is biologically inert and nontoxic. These properties allow its use as a component of bioceramic materials for medical applications [13]. Due to its whiteness, wollastonite is widely used as a pigment for varnishes, paints, enamels, paper, etc., providing white color as well as high strength and antifriction properties.

Previously [14], while studying the optical properties of calcium silicates formed in a CaCl_2_-Na_2_SiO_3_-H_2_O system, it was found that these silicates are characterized by high reflection coefficients at wavelengths up to 200 nm. The whiteness of the samples calculated from their reflection spectra was 95–98%. It has also been shown that wollastonite-based material has high reflectance in visible and near-IR ranges as well as high radiation resistance. Therefore, it may be recommended as a pigment for temperature-controlling coatings of “solar optical reflector” class spacecrafts. It is of interest to obtain and study such coatings based on wollastonite.

Organic binders compared with silicate binders have lower radiation resistance and undergo partial destruction accompanied with gas evolution in outer space conditions [15,16]. Therefore, relatively low-melting lead silicate PbSiO_3_ (mp 760 °C) looks more promising as a binder for reflecting coatings.

Among the most important characteristics of functional coatings is their adhesion to the substrate. There are some different theories of adhesion [17]; among them, chemical bonding, diffusion and mechanical interlocking are of interest. According to the chemical bonding theory, new chemical bonds should form at the interface of the molten coating material and titanium (or titanium oxide). According to the diffusion theory, titanium ions should diffuse in the molten silicate layer, respectively. However, it is known that titanium has high affinity for oxygen, and the oxide layer formed at its surface decreases the adhesion of many materials [18]. This circumstance may cause problems with the formation of functional coatings on titanium and its alloys. Thus, the mechanical interlocking may be the correct way to improve the adhesion. According to this theory, the adhesion depends on the mechanical bond of the coating to the surface. Therefore, the more developed the surface, the better the adhesion gained. 

A method of forming the porous layer on titanium substrate was proposed in [19]. The aim of this work was to study the composition and the structure of the coatings based on a CaSiO_3_-PbSiO_3_ system and to determine the best method for improving the adhesion of such coatings to titanium substrate: the preliminary oxidation, the formation of surface porosity (using the original technique) or the combination. It has been shown that surface porosity allows a significant increase in the adhesive strength of the coating, while pre-oxidation of the surface results in a decrease in the adhesive strength. The results of the study are of interest for developing a decision support system to provide recommendations for setting up control parameters of technological operations.

## 2. Materials and Methods 

The samples of VT1-0 titanium alloy (i.e., technical grade titanium, 99.5 wt.% Ti) with dimensions of 25 × 15 × 2 mm^3^ were used as a substrate. To form a porous surface, two-step treatment was used. At the first step, laser cladding with TiC powder (80–100 μm fraction, ≤1.6 wt.% free carbon) was carried out. Then, the samples were etched in boiling nitric acid (57%, analytical grade) for 30 min to completely remove the TiC phase.

Laser cladding was carried out using a universal robotic system consisting of KR 30-3HA robot (KUKA, Augsburg, Germany), KRC4 control system (KUKA, Augsburg, Germany), DKP-400 positioner (KUKA, Augsburg, Germany) and *LS-1-K* ytterbium fiber laser (IPG Photonics, Oxford, MA, USA) with 100–1000 W continuous power and wavelength equal to 1.070 μm. The parameters of the treatment were as follows: the laser spot radius was 0.6 mm, the step between tracks was 0.5 mm, the distance from nozzles to the substrate surface was 10 mm, laser radiation power was 250 W, travel velocity of the beam was 20 mm/s. The treatment was carried out in a pure argon atmosphere.

Four series of experiments were carried out for estimating the effect of the initial state of the surface on the adhesive strength of coatings. In the first series, the samples with smooth surfaces were used without preliminary treatment. In the second series, the surface of the samples was made porous by cladding with TiC and etching (as described above). In the third and the fourth series, the samples with smooth and porous surfaces were also used, respectively, but after additional high-temperature oxidation (800 °C, 1 h).

To synthesize calcium hydrosilicate, sodium silicate and calcium chloride were used. Sodium silicate was taken as an aqueous solution (contained 22.4 mass.% Si) with silica modulus M = 1 (“chemically pure” grade). Calcium chloride was taken as a dihydrate CaCl_2_ 2H_2_O (≥98.3 mass.% CaCl_2_ 2H_2_O). The reagents were mixed in an aqueous medium and autoclaved at 220 °C for 3 h. The resulting bulk precipitate was washed with distilled water until the Cl^−^ ions were completely removed. The presence of chloride ions was controlled by the reaction of washings with AgNO_3_ solution. After washing, the precipitate was filtered and dried at 85 °C. To obtain a crystalline form of pseudowollastonite, calcium hydrosilicate was annealed at a temperature of 1200 °C for 5 h.

Lead silicate was synthesized by mixing the solutions of sodium silicate (M = 1) and lead acetate Pb(CH_3_COO)_2_ in an open container. Washing and drying were carried out similarly to the synthesis of calcium silicate described above.

The powders of prepared silicates were mixed in a molar ratio CaSiO_3_/PbSiO_3_ = 1/2, uniformly applied onto the surface of titanium samples and heated to a temperature of 800 °C for melting the binder (holding for 1 h, cooling in an oven).

To cut and grind titanium samples for further metallographic research, Micracut 201 precision cutting machine (Metkon Instruments, Bursa, Turkey) and Forcipol 1V grinding and polishing machine (Metkon Instruments, Bursa, Turkey) were used. The heating of the samples to obtain the coatings, the annealing of calcium hydrosilicate and thermal oxidation of titanium samples were carried out in SNOL 6.7/1300 muffle furnace.

X-ray diffaction (XRD) analysis of the samples was carried out on D8 ADVANCE diffractometer (Bruker, Billerica, MA, USA) in Cu K_α_ radiation; X-ray diffraction patterns were identified using the EVA program with PDF-2 powder data bank.

For scanning electronic microscopy (SEM) of the surface, S5500 high-resolution scanning electron microscope (Hitachi, Tokyo, Japan) was used. The microscope was equipped with Thermo Scientific attachment to provide energy-dispersive spectroscopy (EDS) analysis. For optical microscopic analysis, a METAM LV-41 light microscope (LOMO, Saint-Petersburg, Russia) was used. The numerical values of the elements’ content were obtained as the arithmetic mean of 3-5 points on the analyzed surface.

The adhesive strength of silicate coatings with titanium substrate was tested on a Autograph AG-X plus 50 kN tensile machine (Shimadzu, Tokyo, Japan). To carry out testing, a steel cylinder with a diameter of 10 mm was glued with Yonglian epoxy adhesive to a coated surface of samples. Then, the sample and the cylinder were fixed in special clamps and vertical tensile load was applied. The tension was carried out with a speed of 0.5 mm/min. The adhesive strength was calculated using the formula σ_a_ = F/S, where F is the stress causing a detachment of the coating, and S is the area of the coating detached from the substrate. The measurements were carried out according to ISO 4624:2002. In each of four experimental series, the adhesive strength was found as the arithmetic mean for three samples tested. 

## 3. Results and Discussion

### 3.1. Forming Porous Surface Layer

**Figure 1:**
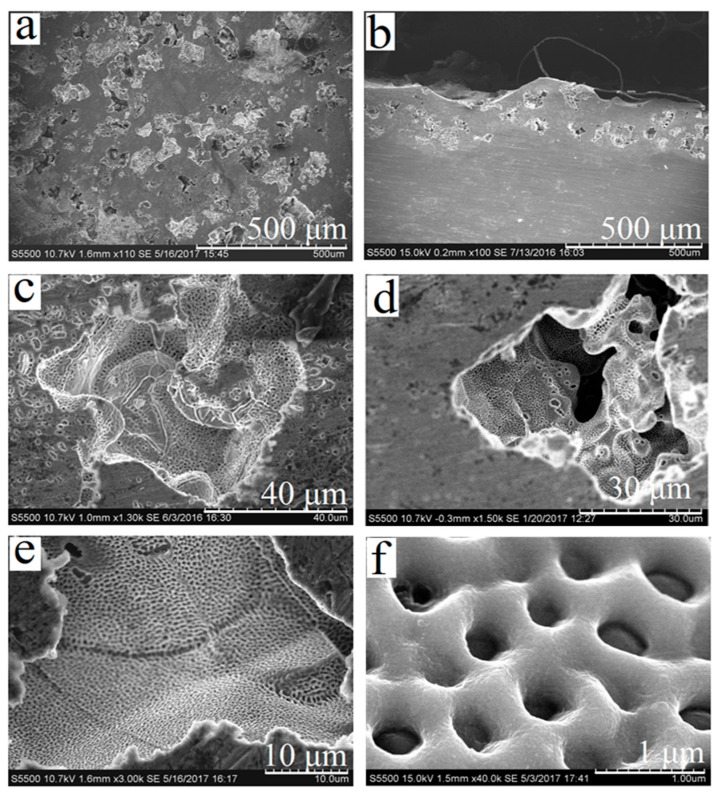
Porous surface of titanium samples after laser glazing with TiC powder (80/100 μm fraction) and etching with nitric acid: (**a**) surface appearance of sample (110×); (**b**) cross-section of sample (100×); (**c**,**d**) individual pores (1300× and 1500×, respectively); (**e**,**f**) inner surface of pores (3000× and 40,000×, respectively).

As a result of laser cladding with titanium carbide powder and subsequent selective etching in nitric acid, surface porosity can be formed on titanium substrate. In [19], it was noted that it is possible to control the size of pores and the thickness of porous layers by changing the processing conditions. At the processing parameters used in this work, the thickness of porous layer was 150–200 μm, and the most typical pore sizes were commensurable with TiC powder fraction—i.e., 80–100 μm (Figure 1a,b). To increase the adhesion of the binder to the substrate, laser treatment mode providing a two-level porous structure was selected. After the treatment according to this mode, a homogeneous network of nanosized pores was formed on the inner surface of micropores (Figure 1 c–f).

### 3.2. Synthesis of Calcium and Lead Silicates

**Figure 2:**
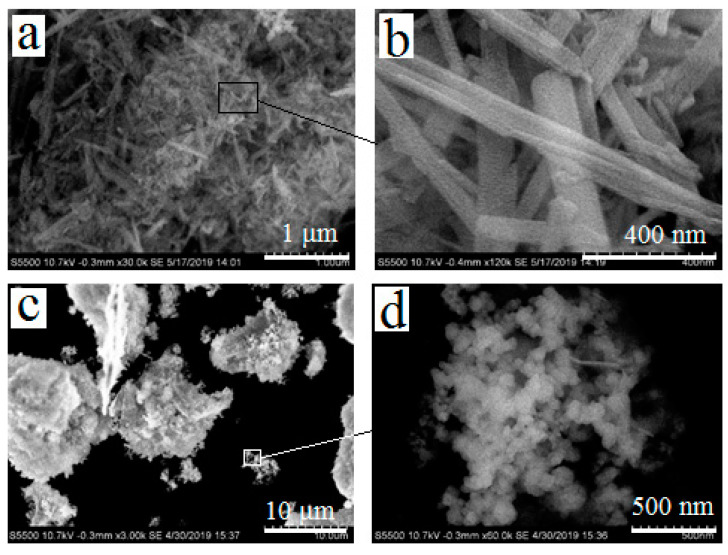
Powders of calcium silicate (**a**,**b**) and lead silicate (**c**,**d**) after drying at 85 °C. Magnifications: 30,000× (**a**), 120,000× (**b**), 3000× (**c**), 60,000× (**d**).

After autoclave treatment of the CaCl_2_-Na_2_SiO_3_-H_2_O system, wollastonite was obtained as needle-shaped crystals (Figure 2a,b). The length of single crystals was about 300–1000 nm, and the width was about 50–100 nm. Since lead silicate melts during coating the substrate, there was no need to obtain it in a crystalline form. Therefore, the synthesis of PbSiO_3_ was carried out without using an autoclave, in open containers, and the product was obtained as an amorphous precipitate (Figure 2c,d).

**Figure 3:**
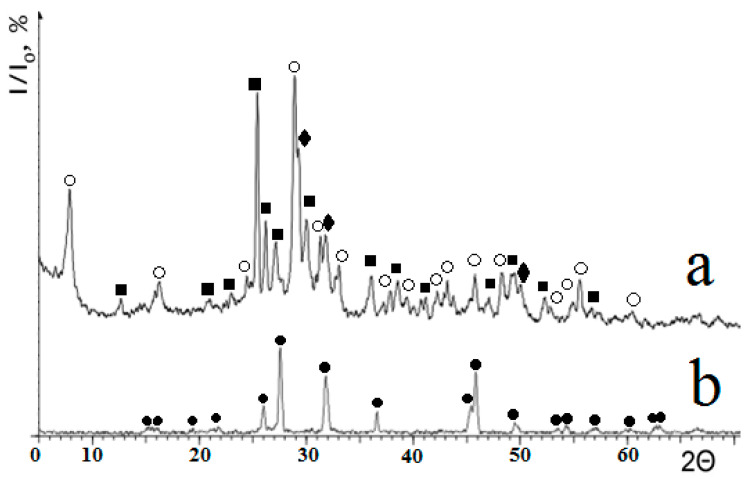
X-ray diffraction patterns of calcium silicate samples: (**a**)—initial CaSiO_3_ sample after autoclave synthesis; (**b**)—after annealing at 1200 °C for 5 hours. Phase designation: ○-tobermorite 9A Ca_4_Si_6_O_15_(OH)_2_·5H_2_O; ■—wollastonite CaSiO_3_; ♦—calcium hydrosilicate Ca_1,5_SiO_3,5_·xH_2_O; ●—pseudowollastonite CaSiO_3._

According to XRD analysis, wollastonite powder contained some aqueous calcium silicates—e.g., tobemorite 9A (riversideite) Ca_4_Si_6_O_15_(OH)_2_·5H_2_O and calcium hydrosilicate Ca_1.5_SiO_3.5_·xH_2_O (Figure 3a). After further annealing of the powder at 1200 °C for 5 h, water was removed and monoclinic pseudowollastonite (PDF-2, 01-089-6463: a—11.83220; b—6.86240; c—10.52970; α = 90.000; β = 111.245; γ = 90.000), i.e. the high-temperature clacium silicate was formed (Figure 3b). 

According to the data obtained in [14], pseudowollastonite has the lowest integral solar absorption coefficient in comparison with other forms of calcium silicate. Therefore, the temperature (1200 °C) and the duration (5 h) of the annealing were chosen to provide complete transformation of wollastonite to pseudowollastonite.

### 3.3. Obtaining and Studying the Coating

**Figure 4:**
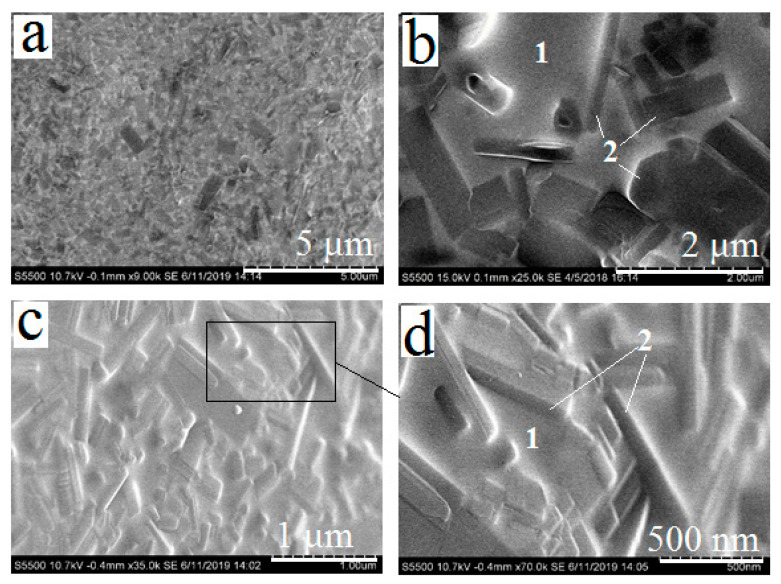
Silicate coating based on CaSiO_3_-PbSiO_3_ system, after sintering (series 1). Magnifications: 9000× (**a**), 25,000× (**b**), 35,000× (**c**), 70,000× (**d**).

After mixing the powders of silicates, the mixture was applied onto titanium samples (series 1) and underwent a sintering (800 °C, 1 h). As a result, PbSiO_3_ melted and formed glass layer binding pseudowollastonite crystals after cooling (Figure 4). The thickness of the layer after cooling was 200–300 μm. A large amount of the amorphous phase was formed in the coating, so it was not possible to determine its phase composition accurately by XRD. The elemental composition of the phases marked as 1 and 2 (Figure 4b,d) was determined using EDS, and the results are presented in Table 1.

Thus, the coating obtained after the sintering comprises needle-shaped pseudowollastonite crystals and a glass matrix. The average sizes of calcium silicate crystals remain the same as before the formation of the coating. Therefore, the mutual solubility of the components seems to be low at the temperature of the sintering. The presence of about 1 at.% of calcium in the composition of the binder may indicate both a measurement error due to the close arrangement of phases and the probability of cation exchange as a result of the interaction of silicate components during the melting of the binder. Lead is also present in the elemental composition of CaSiO_3_ needles (about 3.36 at.%). This is most likely caused by the fact that molten PbSiO_3_ completely covers the needle crystals, and a certain amount of lead is inevitably identified at energy-dispersive analysis.

**Figure 5:**
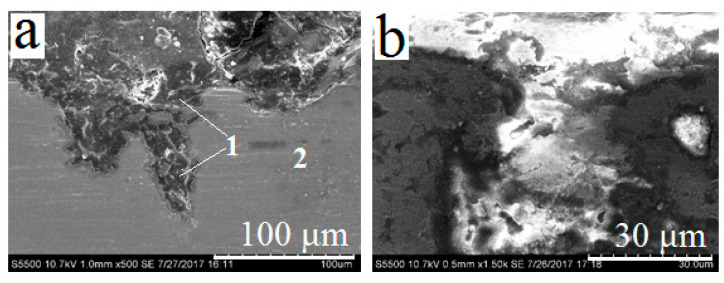
Cross-section of porous titanium samples (series 2) after coating. Magnifications: 500× (**a**), 50,000× (**b**).

When porous titanium samples (series 2) were used as a substrate, the molten lead silicate PbSiO_3_ penetrated the pores. That should increase the adhesion of the coating to the substrate. In order to obtain data on filling pores with silicate mixture, the samples after sintering were cut to prepare cross-section samples (Figure 5). As can be seen in the presented SEM images, the melt densely fills the pore space during sintering, leaving no voids or gas bubbles. The elemental compositions of surface layer and metal substrate are given in Table 2. The presence of carbon in both regions is probably the result of surface contamination during grinding and polishing of cross-section samples.

In addition to the main elements, about 20.4 at.% of titanium was identified in the amorphous binder. Moreover, the highest content of titanium in the coating was observed in the areas directly adjacent to titanium substrate. Thus, silicate components interact with titanium substrate. It was confirmed by the results of XRD analysis that new peaks corresponding to the crystalline phase of lead titanate PbTiO_3_ (makedonite) were identified on the diffraction patterns of cross-section samples. Lead titanate may be formed as a result of an interaction of the molten lead silicate with a titanium dioxide layer presenting on the titanium surface during the sintering, according to the reaction:

PbSiO_3_ + TiO_2_ → PbTiO_3_ + SiO_2_ (1)

Lead titanate is a ferroelectric widely used as a component for piezoelectric ceramics and dielectric coatings [20,21,22]. 

**Figure 6:**
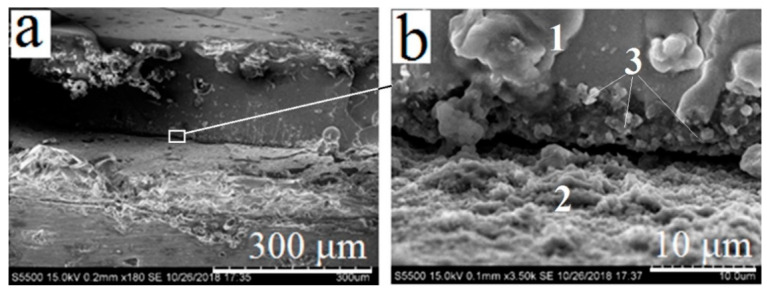
Cross-section of oxidated nonporous titanium samples (series 3) after applying the coating. Phases are marked as follows: 1—glassy PbSiO_3_; 2—TiO_2_ layer; 3—crystallites of PbTiO_3_ and SiO_2_. Magnifications: 180× (**a**), 3500× (**b**).

In series 3, smooth titanium samples were subjected to preliminary thermal oxidation at 800 °C for 1 hour to form an oxide layer before applying the coating. This oxidation was carried out to provide a more detailed study of new phase formation at the interface, as well as to assess its effect on the adhesive strength of the coating to the substrate. In this case, the surface contacting with PbSiO_3_ melt was not the titanium alloy itself but a dense layer of titanium dioxide. The result of the interaction between silicate melt and the titanium dioxide layer can be seen in the SEM images of nonporous titanium samples subjected to preliminary thermal oxidation (Figure 6a,b).

During melting PbSiO_3_ binder, fine-dispersed crystallites are formed at the interface between silicate and oxide layers. The elemental composition of these crystallites, according to EDS, comprises Pb, Ti, Si and O in various ratios. Based on the elemental composition of raw materials and EDS data, the phase composition of these crystallites comprises the products of reaction (1)—i.e., PbTiO_3_ and SiO_2_. In Figure 6b, a delamination of silicate coating from the substrate is clearly visible. The delamination is a result of mechanical action during the grinding of the sample. Obviously, the adhesion strength of the amorphous layer and the substrate decreases with the formation of new crystalline phases at the interface, due to the difference in linear expansion coefficients.

**Figure 7:**
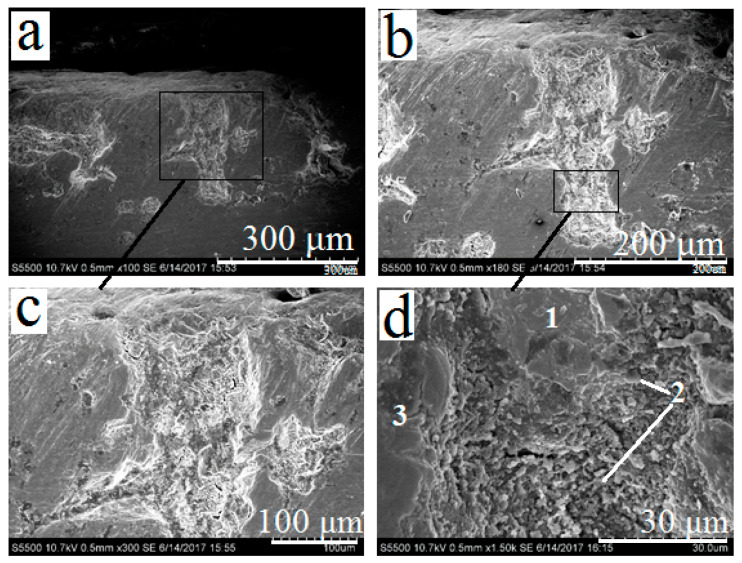
Cross-section of porous oxidized titanium samples (series 4) after coating. Phases are marked as follows: 1—solidified PbSiO_3_; 2—TiO_2_ layer; 3—titanium substrate. Magnifications: 100× (**a**), 180× (**b**), 300× (**c**), 1500× (**d**).

While studying the cross section of porous titanium samples with preliminary oxidation (series 4), the delamination of the coating was not observed. The cross-section of a pore after thermal oxidizing and subsequent coating with CaSiO_3_–PbSiO_3_ composition is shown at Figure 7a. The surface of the titanium sample is covered with a dense layer of TiO_2_. During heating, the molten PbSiO_3_ binder fills surface pores. However, the layer of titanium dioxide presenting inside pores prevents the silicate mixture from completely filling the pore space. Figure 7d shows the lower boundary of the silicate layer which has solidified inside the pore without contacting with titanium substrate. Based on the elemental composition of the areas (Table 3), their phase composition may be assumed: 1—PbSiO_3_-based glass; 2–TiO_2_; 3—titanium substrate. Like in the previous case, the presence of carbon in surface composition may be caused by conditions of preparing the cross-section samples.

On the basis of the data presented in Figure 7 and Table 3, it may be concluded that PbSiO_3_ melt while filling the pores does not interact with the titanium matrix, but reacts with the TiO_2_ layer according to the reaction (1) with the formation of lead titanate at the interface. Therefore, if an excess of TiO_2_ presents at the surface of titanium samples (including pores), it will prevent the molten silicate mixture from contacting with the titanium surface and form crystalline PbTiO_3_ instead. That should have a negative effect on the adhesive strength of the coating to titanium substrate.

**Figure 8:**
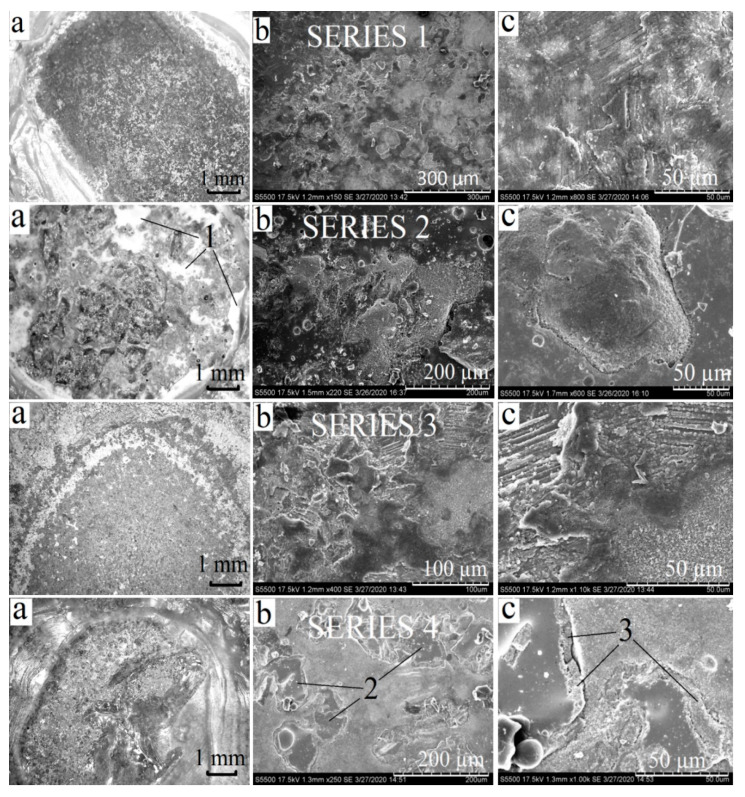
Surface of samples after testing: (**a**) optical microscopy; (**b**,**c**) SEM. Zones are marked as follows: 1—areas coated with remains of adhesive; 2—material of the coating remained in pores; 3—intermediate layer between titanium substrate and silicate coating.

During the adhesive strength tests of the samples with silicate coatings (series 1–4), a destruction of the coating and its delamination from the metal surface was observed in all cases (Figure 8). While applying vertical tensile load to the coatings on smooth titanium samples (series 1, 3), brittle destruction with complete delamination occurred at very light loads. In some cases (while testing pre-oxidized samples), brittle failure of silicate coating happened in the zones adjacent to the place of loading (Figure 8, series 3). That indicates a low level of adhesive strength which is probably caused by the forming of brittle crystalline PbTiO_3_ at the “substrate-coating” interface in pre-oxidized samples. While testing smooth samples without preliminary oxidation (Figure 8, series 1), the coating delaminated completely but only in the area where the steel cylinder was glued to the sample. In these cases (Figure 8, series 1, 3; b, c), SEM images show residual fragments of silicate coating. Therefore, despite low adhesive strength, a delamination of the coating is not strictly determined by the adhesion.

While testing the samples with porous surface (series 2, 4), the fractures of the coating had a combined adhesion–cohesion nature: only a partial delamination of the coating occurred. On porous pre-oxidized samples, a destruction of the coatings was observed at 50–70% of the area subjected to vertical tensile loading (Figure 8, series 4; a). The other part of the coating after the fracture remained as the initial glassy layer without any visible cracks or defects. It can be seen in SEM images (Figure 8, series 4; b, c) that after a delamination of the coating, some silicate material remains in pores. In other words, the material inside pores does not break at the interface with metal substrate but undergoes cohesion-determined failure. At Figure 8, series 4, the intermediate layer between titanium substrate and silicate coating is marked with number 3. As the detailed study has shown, this layer consists of fine crystals of SiO_2_, TiO_2_ and PbTiO_3_ (Figure 6).

While testing porous samples without preliminary oxidation (Figure 8, series 2), a delamination occurred only at 20–30% of the area under the steel cylinder. At the loads of about 6–8 MPa, the glue broke away from silicate layer, but most of the surface being tested remained in its initial state. The destruction of the coating occurred mostly in a cohesion-controlled way, only minor areas of uncoated titanium were observed after testing (Figure 8, series 2; b, c).

**Figure 9:**
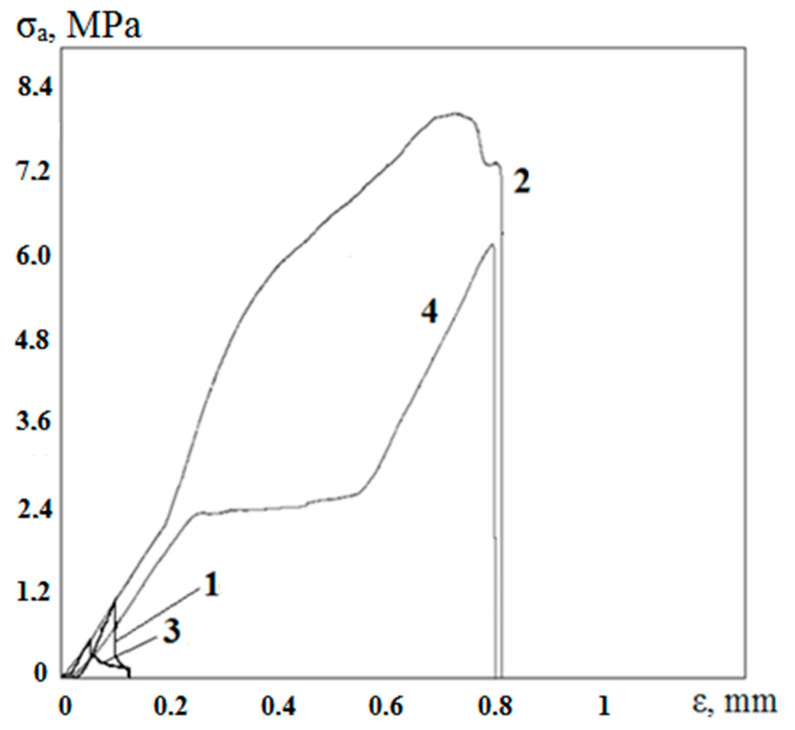
Results of adhesion strength testing (series 1–4).

The results of the adhesive strength testing of coated samples are shown at Figure 9. The average values of the adhesive strength *σ_a_* and maximum deviations for each series are given in Table 4.

The average values of the adhesive strength of silicate coating to porous titanium samples without preliminary oxidation (series 2) reached 8.06 MPa. That is approximately 8 times higher as compared with the initial flat samples (series 1). High adhesion of the coating to porous samples was achieved due to high specific surface area in the place of contact with the molten binder. The same relation of σ_a_ values was also observed while testing samples subjected to preliminary oxidation (series 3 and 4): the adhesion strength of the coating to porous samples (series 4) is by an order of magnitude more than σ_a_ values for flat samples (series 3). Moreover, σ_a_(3) < σ_a_(1), and σ_a_(4) < σ_a_(2)—i.e., the adhesion of the coating to the pre-oxidized surface—was reduced in both cases, as compared with the unoxidized samples. This fact may be explained by the above-described interaction of titanium dioxide film with the molten binder and the formation of the crystalline lead titanate at the coating-substrate interface.

While comparing the results of testing smooth (series 1 and 3) and porous (series 2 and 4) samples, there was not only an obvious increase in the breakaway load of the coating from the substrate but also a significant increase in the ductility of the coating, characterized by an increase in the elongation up to 0.8 mm during testing (Figure 9). Apparently, this is caused by two factors. Firstly, it is the increase in the adhesive strength due to the mechanical bond between the amorphous layer and the developed surface of titanium substrate. Silicate melt, after filling pores and solidifying, provides a significant increase in the measured value of the adhesive strength. As a result, initially, the site of breakdown is not at “coating/smooth substrate” interface but inside titanium substrate, at the “coating/inner surface of pores” interface. Therefore, the process proceeds not as a catastrophic failure but as a step-by-step peeling. On the other hand, epoxy resin adhesive may undergo some deformation during testing. That also could have an effect on test results. Thus, due to the porous surface, not only the high adhesion strength of the coating can be ensured but also a certain margin of ductility.

These results clearly demonstrate the importance of the mechanical bond of the silicate coating to titanium substrate. At the same time, the preliminary oxidation of titanium substrate proved to be undesirable (under conditions of applying the coating in a furnace) since an excess of titanium dioxide on the surface leads to the formation of brittle crystalline PbTiO_3_ at the interface. It is known that technologies of applying coatings for medical applications may include the stage of oxidation (e.g., microarc oxidation) [13]. However, there were no data on if the preliminary oxidation should be carried out before thermal coating.

The point of this study was to evaluate the possibility of forming CaSiO_3_-containing coatings on titanium alloys and the effect of surface porosity on the adhesive strength. Therefore, conventional thermal treatment was used for applying the coating. However, in the future, different methods of forming a coating may be used to improve the quality of the coating and its adhesion to the substrate—e.g., supersonic spraying, plasma spraying, laser surfacing, etc.

Depending on shapes of workpieces and conditions of their use, different techniques and parameters of surface pre-treatment may be required to gain the best result. In this regard, it is important to implement an expert knowledge-based decision support system for automating the setting of control parameters for technological operations of laser powder cladding. Based on the specification of the required result of the technological operation, this system will give recommendations to the process engineer on setting the parameter values of the robotic complex for laser powder cladding.

## 4. Conclusions

Composite coatings containing needle-shaped pseudowollastonite as a filler and lead silicate as a binder were obtained on titanium alloy VT1-0 by applying the powder mixture onto the surface and heating to the melting of the binder. While using titanium samples with a porous surface, filling of the pores with a molten binder is observed, which results in a significant (8–10 times) increase in the adhesion of the coating to the substrate, as compared with nonporous samples. At the same time, an excess of titanium dioxide on the surface (including pores) of titanium samples has a negative effect on the adhesive strength of the coating to the substrate due to the interaction of the molten silicate mixture with TiO_2_ and the formation of brittle crystalline PbTiO_3_ at the interface between the coating and the substrate. 

It should also be noted that the results of this study are of interest for developing a decision support system. This system should be based on a constantly updated and verified base of knowledge on technological operations of coating process. The system will generate recommendations for setting up controlled parameters of technological operations to decrease the number of possible failed experiments before obtaining the required result of the operation.

## Figures and Tables

**Table 1:** Elemental composition of phases marked as 1 and 2 in Figure 4 b,d (at.%).

No. of Area	Ca	Pb	Si	O
1	1.01	22.96	36.61	39.42
2	24.57	3.36	23.93	48.14

**Table 2:** The elemental composition of areas 1 and 2 in Figure 5a (at.%).

No. of Area	Ca	Pb	Ti	Si	O	C
1	1.36	30.12	20.40	8.31	21.16	18.65
2	–	–	80.14	–	1.24	18.62

**Table 3:** Elemental composition of areas 1, 2 and 3 in Figure 6d (at.%).

No. of Area	Pb	Ti	Si	O	C
1	18.39	1.38	22.03	34.79	23.41
2	–	32.81	–	42.96	24.23
3	–	82.71	–	0.21	17.08

**Table 4:** Average values of adhesive strength and maximum deviations (series 1–4).

Series No.	Adhesive Strength σ_a_, MPa	Maximum Deviations, MPa
1	1.06	0.13
2	8.06	0.49
3	0.55	0.09
4	6.2	0.57
